# Integration of the RTS,S/AS01 malaria vaccine into the Essential Programme on Immunisation in western Kenya: a qualitative longitudinal study from the health system perspective

**DOI:** 10.1016/S2214-109X(24)00013-5

**Published:** 2024-02-28

**Authors:** Jenny Hill, Teresa Bange, Jenna Hoyt, Simon Kariuki, Mohamed F Jalloh, Jayne Webster, George Okello

**Affiliations:** a Department of Clinical Sciences, Liverpool School of Tropical Medicine, Liverpool, UK; b Kenya Medical Research Institute/Centre for Global Health Research, Kisumu, Kenya; c Global Immunization Division, Global Health Center, Centers for Disease Control and Prevention, Atlanta, GA, USA; d Disease Control Department, London School of Tropical Medicine & Hygiene, London, UK

## Abstract

**Background:**

Malaria accounts for over half a million child deaths annually. WHO recommends RTS,S/AS01 to prevent malaria in children living in moderate-to-high malaria transmission regions. We conducted a qualitative longitudinal study to investigate the contextual and dynamic factors shaping vaccine delivery and uptake during a pilot introduction in western Kenya.

**Methods:**

The study was conducted between Oct 3, 2019, and Mar 24, 2022. We conducted participant and non-participant observations and in-depth interviews with health-care providers, health managers, and national policymakers at three timepoints using an iterative approach and observations of practices and processes of malaria vaccine delivery. Transcripts were coded by content analysis using the consolidated framework for implementation research, to which emerging themes were added deductively and categorised into challenges and opportunities.

**Findings:**

We conducted 112 in-depth interviews with 60 participants (25 health-care providers, 27 managers, and eight policy makers). Health-care providers highlighted limitations in RTS,S/AS01 integration into routine immunisation services due to the concurrent pilot evaluation and temporary adaptations for health reporting. Initial challenges related to the complexity of the four-dose schedule (up to 24-months); however, self-efficacy increased over time as the health-care providers gained experience in vaccine delivery. Low uptake of the fourth dose remained a challenge. Health managers cited insufficient trained immunisation staff and inadequate funding for supervision. Confidence in the vaccine increased among all participant groups owing to reductions in malaria frequency and severity.

**Interpretation:**

Integration of RTS,S/AS01 into immunisation services in western Kenya presented substantial operational challenges most of which were overcome in the first 2 years, providing important lessons for other countries. Programme expansion is feasible with intensive staff training and retention, enhanced supervision, and defaulter-tracing to ensure uptake of all doses.

**Funding:**

PATH via World Health Organization; Gavi, the Vaccine Alliance; The Global Fund; and Unitaid.

## Introduction

Malaria remains a global public health priority, causing approximately 249 million cases and 608 000 deaths in 2022, with 95% of deaths occurring in sub-Saharan Africa.^1^ Most malaria deaths occur in children younger than 5 years, accounting for more than 450 528 child deaths annually.^1^ Infants and young children in malaria-endemic countries in Africa typically experience several clinical episodes of malaria before they acquire partial immunity, which protects against severe and fatal malaria, and are therefore a high-risk group targeted for malaria treatment and prevention.

Despite impressive gains in malaria control between 2000 and 2015, progress has stalled, and current tools are threatened by drug and insecticide resistance.^2^ Malaria vaccines have the potential to make a substantial contribution to malaria control when used in combination with other effective control measures, especially in areas of high transmission. RTS,S/AS01 is the world's first malaria vaccine to show a protective effect against malaria in young children in a phase 3 trial conducted in seven sub-Saharan African countries (Burkina Faso, Gabon, Ghana, Kenya, Malawi, Mozambique, and Tanzania).^3^ Results from over 4 years of follow-up showed that, among children aged 5–17 months at the time of first vaccination who were given a fourth dose 18 months after the primary series, RTS,S/AS01 reduced clinical malaria by 39%, severe malaria by 31·5%, and malaria-related hospitalisations by 37·2%.^4^ In 2015, the WHO Strategic Advisory Group of Experts on Immunization (SAGE) and Malaria Policy Advisory Committee made a joint recommendation for the pilot introduction and evaluation of RTS,S/AS01 in three-to-five African countries.^5^ The pilots were designed to answer outstanding questions on safety, impact, and feasibility to inform WHO recommendations on the broader use of RTS,S/AS01 in sub-Saharan Africa.

**Research in context**

**Evidence before this study**

We searched PubMed and Medline with no language restrictions using the search terms ((“malaria vaccine” AND “feasibility”) OR (“malaria vaccine” AND “integration”)) AND “children” from Jan 1, 2016, to Dec 31, 2018, and identified five studies. By 2019, there were no published studies on the feasibility of implementation in routine settings. Earlier qualitative studies were conducted in the context of clinical trials or used hypothetical scenarios. A qualitative study exploring sociocultural issues on malaria and vaccines conducted in two malaria-endemic regions of Kenya in 2010 identified concerns about the quality of immunisation services and the need to improve health worker interpersonal communication skills. Another study in Kenya using data from the 2010 Service Provision Assessment Survey found that the likelihood of caregivers accepting their child to receive a malaria vaccine was correlated with province and satisfaction with health-care services, in addition to caregiver factors. During a study using observations of counselling offered to caregivers at clinics in two districts in Ghana, the authors noted that little attention was given to addressing mothers' concerns related to child immunisation. A systematic review of eight studies up to 2017 in sub-Saharan Africa on the challenges faced during malaria vaccine implementation or trials identified inefficient delivery of vaccination services to children and suboptimal quality of the health services. Proposed solutions included use of dynamic communication models and trusted sources for delivering vaccine-related health information to communities, community engagement at national and district levels, and implementation through existing health services. The search was updated on Oct 26, 2023, and identified six new studies. Four studies were conducted in Ghana: two on the challenges and lessons during planning and early vaccine introduction, a post-introduction evaluation of RTS,S/AS01, and an analysis of the determinants of uptake. Two studies estimated the costs of introducing and continuing RTS,S/AS01 malaria vaccination in the three malaria vaccine pilot implementation countries (Ghana, Kenya, and Malawi). Our study sought to explore the contextual and dynamic factors shaping RTSS,S/AS01 integration into the Essential Programme on Immunisation and uptake over an extended period of 2 years covering the full four-dose schedule.

**Added value of this study**

Since the pilot introduction of RTS,S/AS01 in 2019, this is the first longitudinal study to investigate the contextual and dynamic health system factors shaping its delivery and uptake through routine immunisation services. Our study provides an in-depth qualitative analysis of policy maker, health manager, and health-care provider perceptions of the vaccine, and the operational challenges and adaptations made, at each level of the health system and at all stages of implementation, from planning and engagement with stakeholders, through to execution, reflection, and evaluation. Our study found that the integration of RTS,S/AS01 into routine immunisation services presented significant operational challenges, some of which persisted over time, such as intervention complexity and attrition of skilled immunisation staff. Nevertheless, self-efficacy among health-care providers grew as they gained experience of delivering the vaccine. Our findings showed that while the integration of RTS,S/AS01 into immunisation services in western Kenya presented substantial operational challenges, these were largely overcome; however, uptake of the fourth dose remained a challenge.

**Implications of all the available evidence**

Our findings are relevant to the planned expansion of RTS,S/AS01 to other parts of Kenya and to its introduction in other countries in sub-Saharan Africa. Countries can learn from the lessons of the early introduction in Kenya and prioritise funding for well planned and executed implementation that considers intensive sensitisation before implementation, comprehensive training of health-care providers and retention of skills within the workforce, integrated systems for health management information system reporting, assured vaccine supplies, and community-based defaulter tracing systems to ensure uptake of the fourth dose.

In 2019, Ministries of Health in Kenya, Ghana, and Malawi launched the pilot introduction of RTS,S/AS01 in selected areas through routine child immunisation services. The pilots were coordinated by the national Malaria Vaccine Implementation Programmes (MVIP) in each country alongside a WHO-led Malaria Vaccine Pilot Evaluation to provide additional data on safety, the impact on all-cause mortality and severe malaria, and the feasibility of implementing the recommended four doses of the vaccine. Funding for vaccine introduction and selected technical support activities was provided through WHO and the vaccines were donated by the vaccine manufacturer, GlaxoSmithKline.^6^ Our study was part of a larger, multimethod evaluation designed to contribute empirical understanding of the feasibility of the RTS,S/AS01 four-dose schedule requiring new immunisation contacts in the context of the pilot implementation programmes in each of the three countries. Here, we aimed to investigate the contextual and dynamic factors shaping vaccine delivery during the pilot introduction in western Kenya. We present the perceptions and experiences of the RTS,S/AS01 introduction, promotion, delivery, and uptake over time from a health system perspective. Findings from the caregiver and community perspectives are published elsewhere.^7^, ^8^

## Methods

### Study design

**Figure 1:**
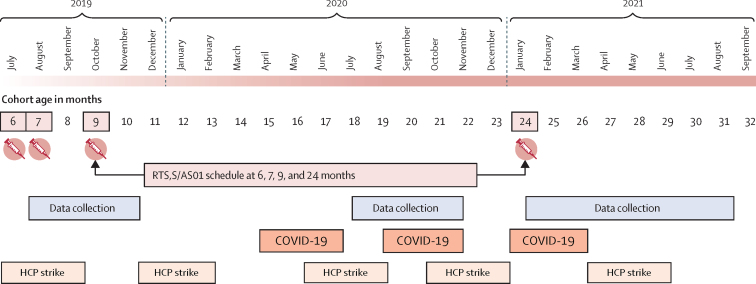
Study timeline in relation to eligibility for RTSS-1 in the cohort and key contextual factors COVID-19 refers to three waves of outbreaks in 2020 and 2021. The first outbreak delayed the second round of data collection. HCP strike refers to HCP strikes affecting the study sites; the first strike at the time of the vaccine launch affected the sites in Kisumu County only. HCP=health-care provider.

Study reporting adheres to the consolidated criteria for reporting qualitative research (COREQ).^9^ The methods are provided here in brief (see appendix 1 p 38 for the study protocol). We undertook a qualitative longitudinal study^10^, ^11^ over a 2-year period following the vaccine launch in Kenya on Sept 13, 2019. We investigated health sector stakeholder perceptions around four key research questions: (1) the challenges and adaptions required to introduce RTS,S/AS01; (2) the perceived impact of MVIP on other vaccination services; (3) the potential for full integration and national scale-up; and (4) the key lessons for deployment elsewhere. Data collection was undertaken at three timepoints (T1–T3) between Oct 3, 2019, and March 24, 2022, designed to coincide with RTS,S/AS01 dose 1 (T1, 2–5 months after introduction), after doses 2 and 3 (T2, 13–16 months after introduction), and soon after dose 4 (T3, 19–26 months after introduction). Figure 1 shows the timeline of data collection in relation to children eligible for the first dose in the initial cohort (aged 6–<12 months) and key contextual factors and events that occurred during the study period, namely the COVID-19 pandemic and health-care provider strikes.

### Study sites

**Figure 2:**
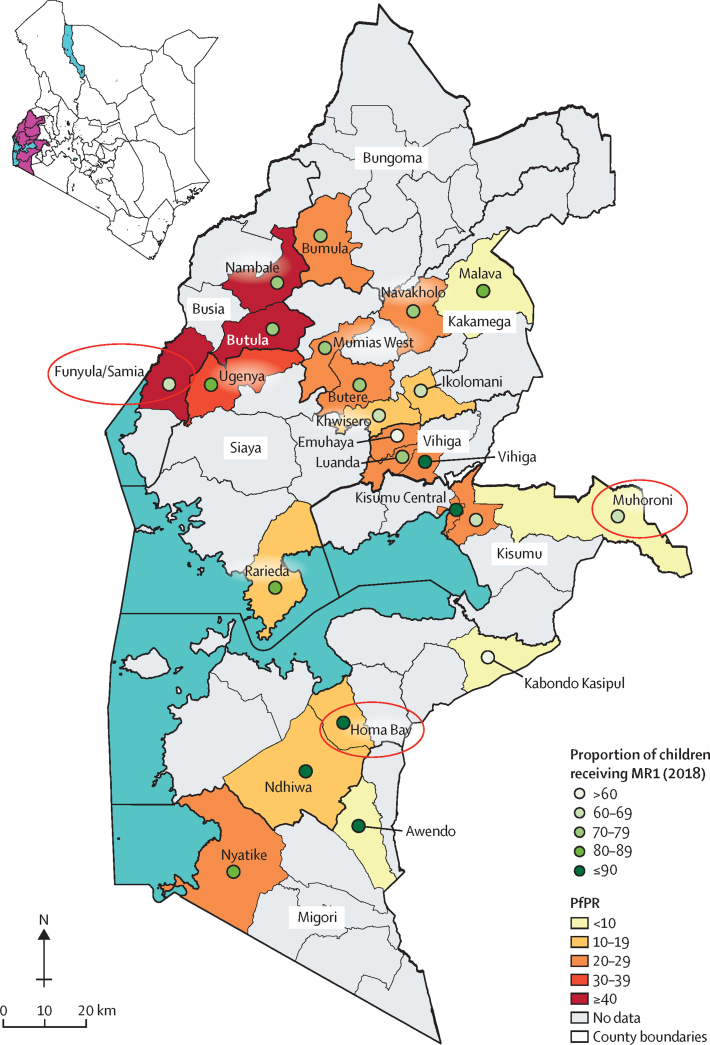
Map of study sites The study subcounties are circled in red, the green dots represent the proportion of children receiving MR1, and the brown shading represents the malaria transmission intensity (see key). MR1=first dose of measles-rubella vaccine. PfPR=*Plasmodium falciparum* parasite rate.

**Figure 3:**
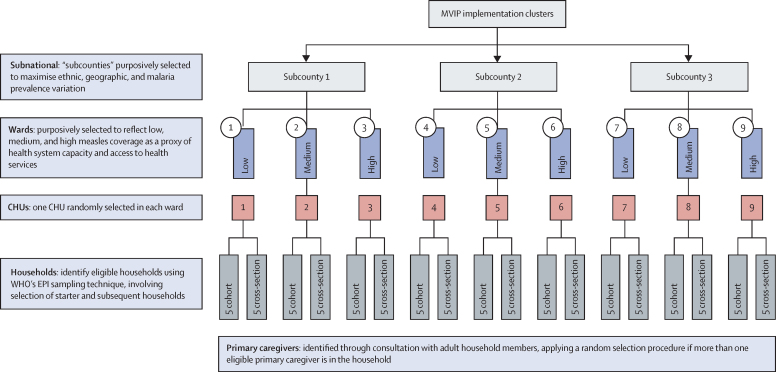
Site selection procedure CHU=community health unit. EPI=Essential Programme on Immunisation. MVIP=Malaria Vaccine Implementation Programmes.

The study was conducted in three subcounties in western Kenya—Funyula, Homa Bay town, and Muhoroni (figure 2). The subcounties were purposively selected from 23 randomly selected RTS,S/AS01 vaccinating subcounties in the MVIP pilot to represent variations in prevalence of *Plasmodium falciparum* malaria among children younger than 5 years (high >40%, medium 10–39%, and low <10%), ethnicity (two Luo-speaking and one Luyha), and geographic setting (two rural and one urban or peri-urban). Within each subcounty, three wards were selected to represent low (<65%), medium (65–75%), and high (>75%) measles vaccination coverage as a proxy indicator for health system capacity and access to immunisation services.^12^ One sublocation was randomly selected from within each ward yielding nine study sites (figure 3). A sublocation is the smallest administrative unit in Kenya, each served by at least one public health facility that is connected to a network of community health units staffed by community health volunteers (CHVs). All health facilities (public, private, and faith-based) providing childhood immunisations in the nine study sites were identified at the study onset, and one facility per site with the highest number of Essential Programme on Immunisation (EPI) clients were purposively selected and enrolled in the study (nine facilities in total).

RTS,S/AS01 was delivered alongside other childhood immunisations in all vaccinating facilities in the pilot areas. The four-dose schedule adopted in Kenya was 6, 7, 9, and 24 months of age. Eligibility for the first dose (dose 1) was subsequently extended to 6–<12 months during the national training of trainers' workshop before the launch in 2019, with doses 2 and 3 given at least 1 month apart, and dose 4 given as soon as possible from 24 months of age, with an upper age limit of 3 years.

### Study population and sampling

We applied an ecological framework approach to identifying study participants with emphasis on the interplay and interdependency between individual, interpersonal, organisational or institutional, and policy levels.^13^ Participants were purposively selected at each level and were the office holders at the time of the interview. At T1, at the facility level, we selected 18 health-care providers (two per facility) responsible for providing vaccination services (table 1). At the management level, we selected 12 subcounty health managers, four from each subcounty health management team (SCHMT) involved in training health-care providers for RTS,S/AS01 introduction, and five county health managers responsible for the coordination of routine immunisation and RTSS/AS01 introduction. At the national level, we identified four ministry of health managers involved in RTS,S/AS01 introduction and one MVIP stakeholder from a multilateral agency, responsible for MVIP coordination in-country. Not all participant groups were included at all data collection timepoints due to feasibility; T2 interviews were restricted to staff engaged in delivering or overseeing vaccination services (ie, health-care providers and subcounty health managers). Participants who were no longer in office in T2 and T3 were replaced (table 1). There were no refusals. We were unable to interview one national health leader in T3 because of unavailability. Participants were first approached by email or telephone, and interviews were conducted in a convenient and private location. Participants provided written informed consent before each interview, except for one participant interviewed virtually who provided informed oral consent. Health-care provider interviews were conducted within the health facilities. Subcounty, county, and national interviews were conducted within the workplace.

**Table 1:** Study participants (sample) and number of interviews by level of health system and timing of interview

	**Description**	**Number of interviews**			
		T1	T2	T3	Total
National health leaders (sample size n=8)	National programme managers for EPI and malaria and other members of the national MVIP technical working group, including research partners and technical agencies	5	..	4*	9
County health leaders (sample size n=7)	From each county health management team: malaria, EPI, and MVIP programme managers	5	..	6†	11
Subcounty health management team (sample size n=20)	Subcounty health management team: malaria, EPI, and MVIP programme managers	12	12‡	14§	38
Health-care providers (sample size n=25)	Heads of health facilities; key staff involved in vaccination and child health programmes (EPI, MCH, IMCI, PHC)	18	18¶	18‖	54
Total (sample size n=60)	..	40	30	42	112

EPI=Essential Programme on Immunisation. MVIP=Malaria Vaccine Implementation Programmes. MCH=maternal and child health. IMCI=integrated management of childhood illness. PHC=primary health care.

* Three new national stakeholders at T3.

† Two county replacements at T3.

‡ Five subcounty replacements at T2.

§ Three further sub-country replacements at T3.

¶ Five health provider replacements at T2.

‖ Two further health provider replacements at T3.

### Procedures

Methods included in-depth interviews and observations (participant and non-participant), and we applied an iterative approach whereby emerging themes or stakeholders of interest were added to subsequent data collection activities. A team of six experienced social science researchers (two male and four female; see Acknowledgements) and a data manager (TB, female) led by a study coordinator (GO, male) collected and processed data continuously for pragmatic coding and analysis, enabling real-time dissemination of findings to relevant stakeholders. GO holds a PhD in Social Science with anthropology and TB holds an MSc in Population Studies and Demography. All staff received training on the principles and methods of conducting qualitative research and study-specific methods before data collection, and refresher training before each interview round. GO conducted all national interviews and observations, and county manager interviews at T1, and the field researchers and data manager conducted all other interviews and observations (at the county, subcounty, facility, and community levels).

Observation data were collected throughout the study by the field researchers. This involved participant observations at national MVIP technical working group meetings and other stakeholder engagement meetings and staff planning, training, and induction meetings at national, county, and subcounty levels, and non-participant observations of vaccine storage, preparation, and administration by immunisation staff in health-care facilities. All participants were briefed about the study and were made aware of our presence in meetings and in health-care facilities. We obtained informed verbal consent from participants to conduct observations.

In-depth interviews were conducted in English, with a target duration of approximately 1 h (range 13–120 min). To ensure data collection was driven by local needs and developments, and to work towards thematic saturation whereby no new themes were identified, the field team kept field notes and held weekly debriefs with GO and TB. After discussion with the Principal Investigator (JHi) and other co-investigators, we iteratively adapted the data collection strategy, identifying new informants or adapting the topic guides. Interviews were audio-recorded unless consent to record was not obtained (one interview), and fieldnotes were maintained on contextual factors and observations.

Development of research materials (topic guides, observation protocol; appendix 1 pp 4–30) was guided by a logic model developed a priori (see protocol, appendix 1 p 31). Topic guides for health-care providers were piloted (two interviews per field researcher in T1–T3) in vaccination areas outside the study communities before data collection began. Basic information about each participant, including their professional role and years of experience, was recorded, followed by open-ended questions addressing topics relevant to each participant group (appendix 1 pp 4–30). Topic guides for national, county, and subcounty levels for T2 and T3 were developed iteratively whereby team debriefings after completion of health-care provider interviews informed topics for the subcounty guides, and debriefings after subcounty interviews informed topics in the county-level and national-level guides.

Participants interviewed provided written informed consent. The study was sponsored by PATH and approved by PATH's Research Ethics Committee, Kenya Medical Research Institute's Scientific and Ethics Review Unit, Liverpool School of Tropical Medicine's Research Ethics Committee, London School of Hygiene & Tropical Medicine's Research Ethics Committee, and the US Centers for Disease Control and Prevention's Center for Global Health.

### Data management and analysis

Audio recordings were transcribed in English, pseudonymised, and transferred to NVivo12 for coding and analysis. Transcripts were first coded by thematic content analysis by JHo using a coding tree that included the health systems building blocks^14^ and themes from the logic model (see protocol, appendix 1 p 31). Coding verification discussions were subsequently held between JHo, JHi, JW, GO, and TB to agree on emerging themes considering contextual factors at each data collection timepoint. Themes were subsequently categorised by JHi using the consolidated framework for implementation research (CFIR; appendix 1 p 34),^15^, ^16^ which addresses the dynamic, multilevel, and transient nature of implementation of interventions in specific contexts. It comprises 39 constructs organised into five domains: (1) intervention characteristics, (2) outer setting, (3) inner setting, (4) individual (patient or provider) characteristics, and (5) process. To explore factors influencing implementation over time we applied trajectory analysis to help preserve chronological flow to understand “what led to what”.^17^ We used time-ordered, sequential matrices, whereby themes from the content analysis were arranged by CFIR construct (y axis) from each timepoint (x axis) using an excel spreadsheet. Factors that impeded or facilitated RTS,S/AS01 delivery were also identified. Observation data and source documents were used to confirm reported events or practices and provide contextual understanding of participants' views. Results were shared and validated with each participant group at dissemination meetings.

### Role of the funding source

The funders had no role in study design, data collection, data analysis, data interpretation, or writing of the report.

## Results

The final sample comprised 112 in-depth interviews with 60 participants (25 health-care providers, 27 managers, and eight policymakers; table 1). Most health-care providers (20 of 25) were female, with an average age of 37 years (range 26–58) and an average number of years working in immunisation of 10 years (range 2–33; appendix 1 p 35). Most providers were registered nurses (17 of 25), and all but five had received training on RTS,S/AS01.

Key themes are presented by time after implementation (T1–T3) supported by panels with quotes for themes by CFIR domain (intervention characteristics, outer setting, inner setting, individual characteristics, and process) and relevant CFIR construct. The dynamics of CFIR constructs over time are summarised in table 2, together with operational challenges and opportunities. Ministry of Health adaptations in response to operational challenges are summarised in table 3.

**Table 2:** Changes in CFIR constructs over time and summary of key challenges and opportunities to RTS,S/AS01 implementation, by CFIR domain

	**T1 post-introduction constructs (2–5 months)**	**T2 post-implementation constructs (13–16 months)**	**T3 post-implementation constructs (19–26 months)**	Challenges (source of data*)	Opportunities (source of data*)
CFIR domain 1: intervention characteristics	Source, evidence, adaptability, complexity, design and packaging, cost	Adaptability, complexity	Evidence, relative advantage, adaptability, complexity	• RTS,S/AS01 schedule is complex and not aligned with existing vaccines (Doc, IDI) • Extended schedule causes low uptake of dose 4 (Doc, IDI) • Temporary adaptations to EPI registers for reporting vaccine indicators affected institutionalisation—eg, HMIS (Obs, Doc, IDI) • Dual vial leads to wastage and provider errors (IDI)	• Evidence of relative advantage in relation to ITN coverage (midline evaluation; Doc, Obs) • Evidence of reduced malaria burden (Doc, Obs)
CFIR domain 2: outer setting	Patient needs and resources	Patient needs and resources	Patient needs and resources	• Limited knowledge of the schedule among caregivers (IDI) • IEC materials produced in English, Kiswahili, and Luo only (Obs) • Children attending from non-vaccinating subcounties (IDI) • Too many doses and fear of side-effects (IDI) • Inadequate social mobilisation and community engagement (IDI, Obs) • Health system constraints—eg, strikes, health provider attitudes (IDI, Obs)	• Widespread vaccine acceptability and demand even in non-vaccinating subcounties (IDI)
CFIR domain 3: inner setting	Compatibility, leadership engagement, available resources, access to knowledge and information	Compatibility, available resources, access to knowledge and information	Compatibility, leadership engagement, available resources	• County (and subcounty) health managers have limited resources for training and supervision (IDI) • Some lingering compatibility issues with reporting tools (IDI, Obs) • Discrepancy between RTSS schedule in the guidelines and the schedule in reporting tools (IDI, Obs)	• Regular national MVIP coordination meetings and strong MVIP leadership (IDI, Obs) • Subcounty managers devised alternative systems to support health providers (eg, WhatsApp) to provide information and guidance (IDIs) • Institutionalisation issues largely overcome (IDIs, Doc, Obs) • Political will for scale up to non-vaccinating sub-counties (Obs, Doc)
CFIR domain 4: characteristics of individuals	Knowledge and beliefs about the intervention, individual state of change	Self-efficacy, individual state of change	Self-efficacy, individual state of change	• Attrition of skills due to staff rotation between vaccinating and non-vaccinating clusters or departments (IDI, Doc) • New staff lack RTSS training and skills (IDI)	• Self-efficacy increased over time as health-care providers acquired experience and confidence to give the vaccine (IDIs)
CFIR domain 5: process	Planning	..	..	• National MOH lack of control over WHO funding (IDI) • Rushed launch compromised quality (IDI) • Issues with schedule adjustments made during training and non-alignment with the distributed reporting tools and IEC materials (IDI, Obs, Doc) • Temporary adaptations to EPI registers and reporting systems increased workload and resulted in discrepancies (IDI)	• Financing and vaccine externally sourced or provided (IDI, Doc)
CFIR domain 5: process	Engaging	Engaging	..	• Limited stakeholder engagement with county stakeholders (IDI) • Lack of CHV engagement and training (IDI, Obs)	• Strong technical partnership of MOH, WHO, and partners (IDI, Obs) • Strong national champion in MOH (IDI, Obs)
CFIR domain 5: process	..	Executing	Executing	RTS,S/AS01 specific: • Lack of health provider supervision (IDI) • Unclear guidance on children presenting off-schedule or from outside a vaccinating subcounty leading to non-standardised approaches by facility—ie, either given or withheld (IDI, Doc) General health system: • Health worker strikes (Obs, IDI, Docs) • Health provider attitudes (IDIs) • Lack of SAE reporting on vaccines (IDIs, Doc) Contextual: • COVID-19 restrictions led to changes in service provision—eg, group health talks ceased; counselling either given one-to-one or not at all (IDIs, Obs) • Population movements due to COVID-19 leading to defaulting (IDIs, Obs)	• Strong national leadership (IDI, Obs) • Ongoing pilot evaluation provided findings in real time to inform managers of programme challenges so that strategies to improve delivery could be deployed (Obs, IDI) • County where CHVs were actively engaged achieved higher coverage (Doc) • Mixed strategies used to capture defaulters—eg, phone call reminders by health providers or CHVs to mothers; health provider reminders at next immunisation visit; outreach; RTSS delivery combined with MR2 catch-up campaign (IDI)
CFIR domain 5: process	..	..	Reflecting and evaluating	• Adjustment to the RTS,S/AS01 regimen to give the fourth dose at 18 months, alongside MR2 (IDI) • Insufficient involvement of county-level focal persons and a need to strengthen county health management team capacity (IDI) • Continuous engagement and training for health providers (IDI) • Operational costs will increase for scale-up (IDI)	• No major safety concerns (Obs, Doc) • Evidence on efficacy of the fourth dose forthcoming to inform RTS,S/AS01 schedule (Obs, Doc)

CFIR=consolidated framework for implementation research. CHV=community health volunteers. Doc=documentation. EPI=Essential Programme on Immunisation. HMIS=health management information system. IDI=in-depth interview. IEC=information education and communication. ITN=insecticide-treated nets. MOH=Ministry of Health. MR2=second dose of measles and rubella vaccine. MVIP=Malaria Vaccine Implementation Programme. Obs=observation. SAE=severe adverse event.

* Data sources were either Obs, Doc, or IDI.

**Table 3:** Key operational challenges identified by participant group and health sector response or adaptations by T3

	**Participant group**	**Response or adaptation made**
**Intervention characteristics**		
1. RTS,S/AS01 dosing schedule	HCPs and CHLs	Job aid (only became widely available by T3)
2. CHVs not trained on the vaccine	HCPs, CHLs, and NHLs	Not fully addressed
**Inner setting**		
3. Substantial knowledge gaps	HCPs and CHLs	OJT, peer support, external agent (KEMRI staff), trying to keep staff transfers within implementing sub-counties
4. Substantial knowledge gaps	SCHMT and CHLs	WhatsApp used by SCHMT across the three subcounties and respective facility staff
5. Health provider strikes	All participants	Following up defaulters, updating register if infant was vaccinated at a different or private facility
6. Lack of supportive supervision	HCPs, SCHMT, CHL, and NHLs	Not addressed at county and subcounty levels; supervision limited to national new vaccine post-introduction evaluation and periodic intensification of routine immunisation surveys
7. Lack of Luhya IEC materials	Ethnography	HPs use of a translator, CHVs were also active in this county and use local language when conducting health education activities
**Outer setting**		
8. Children attending from non-vaccinating clusters	HCPs, SCHMT, CHL, and NHLs	Mixed response by facility (RTS,S/AS01 given if caregiver considered able to complete the course for her child, or withheld; CHLs communicated with non-vaccinating subcounties to await national rollout)
9. Children attending off schedule	HCPs, SCHMT, CHLs, and NHLs	Mixed response by facility (RTS,S/AS01 given or withheld)
10. Non-adherence or LTFU	HCPs	Mixed strategies—eg, phone calls to mothers by HCPs; phone calls to mothers by CHVs; reminders by HCP at next EPI visit; outreach; RTSS delivery combined with MR2 catch-up campaign (June 2021)
11. COVID-19 restrictions	HCPs	Group health talks ceased; counselling either given one to one or not at all
**Process**		
12. Insufficient number of staff trained	HCPs, SCHMT, and NHLs	Not fully addressed
13. Insufficient community awareness prior to launch	HCPs, SCHMT, and CHLs	Insufficient resources though some radio spots and health promotion activities were reported
14. Temporary adaptations to EPI registers and systems for HMIS reporting	HCPs and SCHMT	New integrated permanent registers printed and disseminated to all immunising facilities (only became widely available by T3)

CHL=county health leader. CHV=community health volunteers. EPI=Essential programme on Immunisation. HCP=health-care providers. HMIS=health management information system. KEMRI=Kenya Medical Research Institute. LTFU=lost to follow-up. MR2=second dose of measles and rubella vaccine. NHL=national health leader. OJT=on-job training. SCHMT=sub-county health management team.

The vaccine launch in Kenya, which had originally been planned for April, was delayed by 5 months and occurred in September. Although national health leaders considered themselves to be the main decision-makers for malaria vaccine implementation, they reported limited control over accessing WHO funds for preparatory activities. T1 interviews revealed initial leadership challenges between the national malaria and vaccine programmes over the malaria vaccine pilot. There were also some initial tensions between the WHO-Kenya Office and the Ministry of Health in terms of leadership and ownership of the MVIP programme and alignment of priorities. Participants had differing perspectives on the success of advance preparations. The major issue for national stakeholders was access to WHO funding. While health-care providers generally felt prepared and believed communities to be sufficiently well sensitised, county and subcounty leadership felt the launch had been rushed.

The context of vaccine introduction, as explained by national policy makers at T1, was as a pilot implemented alongside a WHO-led evaluation, with the malaria vaccine donated by GlaxoSmithKline, and the MVIP programme funded by WHO. While government officials described the pilot as “phased implementation”, the perception among health managers and health-care providers was that of a study since it involved randomisation of subcounties into vaccinating and non-vaccinating subcounties (panel 1).

Panel 1 **Themes related to Intervention characteristics—source and evidence**

Respondent (R): “And I think that is where the mistake was. There was so much focus on it being a study rather than it being introduced through the routine system.”

*National health leader*

R: “So, I believe it should be introduced in the other subcounties so that we avoid this issue of thinking that it is a study and people are thinking that they are guinea pigs.”

*County-1 health manager*

R: “So, some of them normally say that they were kind of discriminated and that is politics. But bearing in mind that we have got two subtribes in this county some people were thinking that why was it not taken to the other subcounties and only brought to these ones? So, it has brought some little bit of political issues.”

*County-2 health manager*

Randomisation also brought a sociopolitical dimension to vaccine introduction and misconceptions about the purpose and intent of phased introduction. Health-care providers reported receiving little guidance on how to manage demand from non-vaccinating subcounties (panel 2).

Panel 2 **Theme related to outer setting—patient needs and resources**

Respondent: “at first there were some people also coming from outside the implementing subcounties so they would have some numbers from, let's say subcounty X is doing it, you would get cases of people that are coming from subcounty Y [non vaccinating] area because they are neighbouring, and it called for a lot of wisdom from our people”.

*County-3 health manager*

After the launch, participants were generally positive about the vaccine and its value in the malaria control arsenal, although health-care providers wanted more information about its protective effect*.* County health managers and health-care providers shared concerns about the extended schedule into the second year of life and the poor alignment of the four-dose schedule with other child health services, requiring new clinic visits (panel 3). Health-care providers acknowledged that additional effort would be required to ensure uptake of all doses, placing importance on defaulter tracing. Additional concerns related to the multidose vial, and providers expressed preference for a single-dose vial to avoid wastage and to reduce potential for provider errors when reconstituting the vaccine. They were also concerned that caregivers might find the number of doses burdensome, given that they already complained about too many childhood vaccinations.

Panel 3 **Themes related to Intervention characteristics—adaptability and complexity**

Interviewer (I): “What are other challenges to delivering all the four doses of the malaria vaccine?”Respondent (R): “Number one is that issue of keeping track because these clients are used to when they (their children) are injected with the 9 months vaccine or when done with the vaccines that are administered on the thighs then they think that their child is (fully) vaccinated. So, coming back only for malaria vaccine I think they have not considered it to be of great importance...The duration between the third dose and the fourth dose will facilitate the loss of these clients. I wish it was possible for all the four doses to be consecutive so that they finish them all and they are good to go.”

*Community-14 health-care provider (BSc nursing)*

I: “And then finally what about the eligibility for dose 4, what challenges have you encountered about this?” R: “You know the major challenge that we encountered is that it was not clear when we left the training because at the training, we were told that the fourth dose is 15 months after the third dose. So in other scenarios we used to…somebody got the second dose after 1 month, and the third dose after 1 month also now we were giving them a TCA [return date in child health booklet] for the fourth dose after 15 months from the third dose. But later it was corrected that once they turn 2 years, and 1 month has elapsed since they got their third dose, they are good to get the fourth dose.”

*Community-10 health-care provider (registered nurse)*

I: “Have you had any challenges specific to eligibility for doses two and three?” R: “We only had for dose 4, which was changed from 24 months to 4 weeks now. I mean at a frequency…between dose 1 and dose 2, 4 weeks, and dose 3 and 4, 4 weeks also.” I: “And then 3 and 4 is no longer 15 months?” R: “Yes, you can give at 24 months or after 4 weeks.”

*Community-12 health-care provider (registered nurse)*

Following the pre-launch national trainer-of-trainer training workshop, the subsequent trainer-led cascade training at health facility and community levels was not done as planned because of insufficient resources, according to county and subcounty managers. During the national training workshop, a change was made to the vaccine schedule (observation), extending eligibility from 6–<12 months of age. This new schedule did not align with the pre-printed and distributed documentation tools containing the original eligibility criteria, which were the main source of vaccine information in the absence of cascade training. Health-care providers and managers reported that on-the-job training was not always realised, resulting in widespread uncertainty about eligibility and how to deal with children who were brought off-schedule or from non-vaccinating subcounties in addition to some untrained providers giving malaria vaccinations. This situation was said to be exacerbated by the lack of resources for monitoring and evaluation activities according to county managers, and by the lack of resources for supervision, according to subcounty staff. Budgetary constraints were also responsible for limited community engagement including with CHVs. National stakeholders blamed the inability of the Ministry of Health to access WHO funding to train and supervise CHVs on community mobilisation and defaulter tracing as a major constraint to vaccine uptake.

A key health system compatibility issue identified after the launch concerned the reporting of malaria vaccine indicators. These indicators were added as a temporary adaptation to EPI registers, and health-care providers complained reporting these data created additional workload. County health managers noted this additional burden led to some erroneous data entry.

After a year of implementation (T2 interviews), while the Ministry of Health had printed and distributed new EPI registers containing malaria vaccine indicators, not all health-care providers had received them, and the new registers were not available in private facilities. Providers said their vaccine confidence had been boosted by the progress in the uptake of doses 1–3. They believed that adherence to dose 4 was achievable even though they were concerned about low community awareness of the later doses despite provision of group or one-to-one health education and efforts to record return dates in child health booklets. Additional concerns from providers were that side-effects led some caregivers to delay or avoid bringing their child for their next dose.

The COVID-19 pandemic brought additional challenges, such as mask requirements, social distancing preventing group education, migration out of vaccinating clusters, and lockdown measures, all of which required changes to the way health services were delivered before COVID-19. A series of national and county-level health worker strikes also impacted on vaccine delivery.

2 years after vaccine introduction (T3 interviews), initial expectations of vaccine efficacy appeared to have been realised, as both health managers and providers noted a reduction in the frequency and severity of malaria cases. Providers reported growing community acceptance of the vaccine, which they attributed to increased caregiver confidence in vaccine safety and the lived health benefits in their child. However, concerns had emerged about the low uptake of dose 4 due to low awareness of the vaccine schedule. They noted that caregivers perceived their child to have finished all vaccinations once they reached 18 months, and they highlighted a need for additional strategies such as defaulter tracing and recording return dates in child health booklets (panel 4).

Panel 4 **Theme related to outer setting—patient needs and resources**

Respondent (R): “It is because to some mothers, when the baby has reached 1 year or 18 months, they feel that they are through with the injectable vaccines but would just be going for the Vitamin A and Measles 2, which is given at 18 months...” Interviewer (I): “How many mothers do you see coming at 18 months for weighing and Vitamin A?” R: “Very minimal…, most of them do not come.” I: “Do you think this will affect the fourth dose of the malaria vaccine?” R: “Yes, it will.”

*Community-17 health-care provider (registered nurse)*

There were structural barriers to vaccine access and uptake, such as migration away from vaccinating subcounties during COVID-19, particularly in Homa Bay, and cross-border movement in Funyula on the Kenya–Uganda border, as well as transport costs, poor roads, distance to the health facility, bad weather, etc. Participants described other general health system weaknesses, such as vaccine stockouts, health-care worker strikes, and poor health-care provider attitudes, as discouraging caregivers from attending health services and contributing to defaulters.

Operational challenges had for the most part been resolved by T3 interviews. The Ministry of Health had introduced new guidelines on the vaccine schedule, though not all health-care providers reported having them. Subcounty managers in each subcounty reported devising alternative systems to support providers (table 3). Additionally, managers tried to keep staff transfers within vaccinating subcounties to avoid attrition of skills. Self-efficacy (individual belief in their own capacity to deliver the vaccine) among health-care providers also improved as they overcame many of the operational hurdles and became familiarised with the schedule. Where a national-level response to operational challenges was not implemented, some effective local adaptations were made, including seeking guidance from the MVIP evaluation teams on eligibility and the schedule. The impact of persistent health-care strikes was mitigated through defaulter tracing using mixed strategies. The impact of COVID-19 was tackled by offering daily immunisation services to avoid overcrowding at health-care facilities. The localisation of these adaptations might have led to inconsistencies in vaccine provision across sites.

The vaccine programme was widely viewed to be going well at T3. National health leaders reported good political will and leadership was said to be working well, although they were yet to identify which department would remain responsible for vaccine implementation within the Ministry of Health. In relation to vaccine expansion, a national leader observed “WHO is the technical lead in terms of health and will always be there… working closely with GAVI…they will play an influential role in terms of ensuring that funds are availed”. Overall, national leaders felt the vaccine launch was successful in creating demand, feasibility of integration within the routine system was established, and that coverage was promising. They had no major safety concerns and reserved judgement on the usefulness of dose 4 until more data were available. Leadership at county and subcounty levels, however, continued to be undermined by the lack of resources (panel 5). Planning for national rollout was said to be ongoing, including subnational stratification and consideration of regulatory issues, funding, cold chain, etc, noting that operational costs will increase.

Panel 5 **Themes related to process—reflection and evaluation**

Respondent: “I think in future new introductions there should be elaborate engagement with the counties so that at their level they set aside resources to facilitate the teams to be able to do monitoring of the new vaccines.”

*National health leader*

CHVs had still not been trained, and resource constraints for training new staff and for supportive supervision remained 2 years after the launch. Funyula, which has an active CHV programme supported by a non-governmental organisation, had the highest uptake of dose 4, demonstrating the value of CHV involvement (document and observation data). Health managers and health-care providers cited eligibility confusion, lack of CHV engagement, and inadequate caregiver awareness as the main reasons for low coverage of dose 4 (panel 6). Health managers surmised need for stronger community information and awareness campaigns, comprehensive training of health-care providers and CHVs, positive health-care provider attitudes, and supervision.

Panel 6 **Theme related to inner setting—available resources**

Respondent: “they forgot to bring on board the community health workers who do the defaulter tracing and follow up on any issues in the community. They were just brought on board last week when we were sensitising them. Had they been involved from the beginning; I believe our performance would be better.”

*County-1 health manager*

Malaria vaccine introduction was said to have had a positive impact on uptake of the second measles dose and vitamin A and strengthened growth monitoring visits for children in the second year of life. Both health managers and health-care providers proposed an adjustment to the vaccine schedule to align dose 4 with the second measles dose at 18 months to improve uptake given the coverage for the second measles dose was consistently higher than the coverage for RTS,S/AS01 dose 4 (panel 7).

Panel 7 **Themes related to process—reflection and evaluation**

Interviewer (I): “…what do you think would have been done differently in the introduction of the malaria vaccine?” R: “Mine is just on the fourth dose I think it should be given with measles I think the fourth dose will have high numbers.” I: “Okay, so you suggest that the fourth dose should be given at [?].” R: “18 months if you give it at that time I think the uptake will be very high.”

*Community-13 health-care provider (registered nurse)*

## Discussion

Our study describes the key challenges in the introduction and integration of RTS,S/AS01 through EPI and provides important insights for its expansion in Kenya and deployment in other countries. Challenges were dynamic and changed over time. Although many challenges were resolved with time through adaptations, issues related to intervention complexity persisted 2 years after its introduction, suggesting RTS,S/AS01 is not easily integrated into routine EPI. Health-care provider access to knowledge and information was a major limitation in the first year but subsequently improved as knowledge and experience became institutionalised. Provider confidence in the vaccine and its delivery (self-efficacy) increased as they gained experience; however, low uptake of dose 4 remained a challenge. Patient (ie, caregiver) needs and resources were relevant throughout, as would be anticipated with any new, complex intervention. Collateral operational costs of introduction were not adequately considered or funded, despite the external support from WHO and the vaccine donation from GlaxoSmithKline and, as health leaders noted, these costs will increase with expansion. As Kenya expands the vaccine to non-vaccinating subcounties, more emphasis is needed on supervision, community mobilisation and education, and active defaulter tracing to promote adherence to four doses. Realignment of dose 4 with the second dose of measles at 18 months of age^18^ might improve adherence, as reported in Ghana.^19^ Operational considerations to improve adherence to dose 4, while consistent with WHO guidance,^5^ need to be balanced with the duration of protection.^18^, ^20^

Although previous studies of new vaccine introductions in low-income and middle-income countries (LMICs) found little impact on elements in the health system, except around the time of introduction,^21^ this was not the case in our study. The extent to which the malaria vaccine was integrated in EPI was linked to its phased introduction alongside the nested MVIP evaluation, with commensurate absence of direct control from the Ministry of Health over external funding. MVIP was perceived to be a study, and health-care providers were poorly equipped to respond to unexpected demand from non-vaccinating subcounties. Challenges relating to the nature of RTS,S/AS01 implementation as a pilot were also reported by health managers and providers during RTS,S/AS01 introduction in Ghana.^22^ Inadequate sensitisation and training, and challenges with the vaccine schedule and eligibility criteria, were similarly identified.^22^ Community education on immunisation for children in the second year of life is needed, as identified in Ghana.^23^ The need for more intensive community sensitisation before implementation is essential to optimise vaccine uptake. A systematic review found that use of dynamic communication models and trusted sources for delivering RTS,S/AS01-related health information to communities was an important factor.^24^ Other operational challenges were related to contextual factors, such as the COVID-19 pandemic, which required substantial changes to service provision and increased population mobility, and to general health system weaknesses, such as health-care provider strikes and limited resources for supervision. Regular onsite supportive supervision was identified as a crucial enabler for successful introduction of RTS,S/AS01 in Ghana, suggesting this should be a funding priority for RTS,S/AS01 expansion in Kenya.^25^

The malaria vaccine introduction in Kenya was believed to have perceived benefits on other EPI vaccinations and child health services. The additional contacts provided an opportunity for defaulters to receive missed vaccinations and specifically the second dose of measles. The fact that children were coming for dose 4 was also thought to have improved vitamin A uptake and growth monitoring in the second year of life. In a study of new vaccine introductions in six LMICs, despite facility respondents perceiving that the introductions had increased coverage of other vaccines, the routine data showed no change,^21^ suggesting that these reports from our study should be verified. National stakeholder involvement in generating nationally relevant evidence on the feasibility of vaccine by MVIP partners might have also played a role in vaccine confidence and facilitated integration of the vaccine into routine immunisation, as observed in Ghana.^26^

Our study provides lessons for other countries planning to introduce RTS,S/AS01 or other malaria vaccines, including the recently WHO-recommended R21/Matrix-M vaccine. Leadership at the national level needs to be clearly articulated before vaccine launch, including details of formally appointed leaders, champions, and external change agents, leadership and management arrangements, and mechanisms for interdepartmental collaboration between national immunisation and malaria programmes. Strong leadership will not only build institutional trust but also community trust in the vaccine. Leadership at subnational levels is equally important and requires adequate resources to enable technical oversight and quality assurance, and community engagement. Resource mobilisation should consider the actual costs of vaccine introduction, including collateral costs, as noted in a study conducted for SAGE.^27^ The economic costs per RTS,S/AS01 dose have been estimated to be between three and five times higher than the financial costs owing to the procurement costs of the vaccine.^28^ Costs will need to consider provision of comprehensive pre-service and in-service training and supportive supervision to accommodate a mobile health workforce would mitigate attrition of skilled workers, together with clear and accessible guidelines which tackle the complex vaccine characteristics (packaging, number of doses, and schedule) in addition to guidance for dealing with delayed or off-schedule visits. Robust defaulter tracing mechanisms will also be needed to optimise uptake of the 4-dose schedule. Inclusion of CHVs would facilitate new contacts with caregivers, in addition to engagement with community groups and religious leaders. Malaria vaccine indicators should be integrated into permanent EPI registers and disseminated before rollout. Any subnational stratification of vaccine delivery, for example due to limited vaccine supply,^29^ should consider the potential politicisation that might result and be accompanied by a clear communication strategy.

The strength of our study is that it provides a longitudinal perspective enabling an exploration of the dynamics of the malaria vaccine introduction over time. Evaluation over a longer period meant that it was possible to see how some of the initial implementation challenges were overcome. Our findings should, however, be interpreted in the context of frequent interactions between the research team and the study participants involved in interviews and observations at three timepoints, such that findings could reflect a greater awareness of RTS,S/AS01 than would be anticipated among health-care providers within a routine setting. In addition, it is possible that this interaction with health-care providers could have led to social desirability bias during interviews as the interviewers became known to them. To mitigate bias, we triangulated data from health-care provider interviews with data from other sources, including observations and record review. We also triangulated data with participants at county and national levels with whom the team had less frequent interactions, and with members of the study communities. Selection of health facilities with higher EPI client numbers mean that the perceptions and practices of health-care providers might not represent providers working in facilities with lower client numbers.

In conclusion, our findings revealed that widespread vaccine confidence and increased self-efficacy among health-care providers as well as strong technical and programme leadership support the potential for successful integration of RTS,S/AS01 in routine EPI and expansion in Kenya. Expansion to other parts of Kenya will require intensive training and retention of skilled staff, supervision, targeted and effective community communication, and CHV defaulter tracing to improve uptake of dose 4. Key lessons for deployment elsewhere include strong leadership and interdepartmental cooperation, extensive pre-launch planning, training and community sensitisation, supportive supervision, and consideration of intervention complexity.

## Equitable partnership declaration

## Data sharing

The data sharing agreement is available in the appendix (p 31).

## Declaration of interests

We declare no competing interests.

## Supplementary Materials

Supplementary appendix 1

Equitable Partnership Declaration
