# Supplementary material for: Integration of the RTS,S/AS01 malaria vaccine into the Essential Programme on Immunisation in western Kenya: a qualitative longitudinal study from the health system perspective
Source: Lancet Glob Health. 2024 Feb 28;12(4):e672–84. doi: 10.1016/S2214-109X(24)00013-5 (PMC10932755; doi:10.1016/S2214-109X(24)00013-5)
Supplement: Equitable Partnership Declaration [file mmc2.pdf]

THE LANCET
Global Health

Supplementary appendix 2

This Equitable Partnership Declaration (EPD) was submitted by the authors, and we reproduce it as supplied. It has not been peer reviewed. *The Lancet*'s editorial processes have not been applied to the EPD.

Supplement to: Hill J, Bange T, Hoyt J, et al. Integration of the RTS,S/AS01 malaria vaccine into the Essential Programme on Immunisation in western Kenya: a qualitative longitudinal study from the health system perspective. *Lancet Glob Health* 2024; published online Feb 28. https://doi.org/10.1016/S2214-109X(24)00013-5.

Equitable Partnership Declaration questions

This Equitable Partnership Declaration is a statement being published online alongside papers at *The Lancet Global Health*, as a separate appendix, to allow researchers to describe how their work engages with researchers, communities, and environments in the countries of study. This is part of our broader goal to decolonise global health, handing control and leadership of research to academics and clinicians who are based in the regions of study, and to affected communities.

Please answer all questions with as much detail as possible, noting that all included information will be published open-access and it will be freely available online to all who wish to read it. If a question does not apply to your study, please state "Not applicable".

The format of and questions in this statement are currently in a pilot phase. Please email Dr Liam Messin (Liam.Messin@lancet.com; deputy editor) and Dr Kate McIntosh (Kate.McIntosh@lancet.com; senior editor) with any feedback, particularly if you find any questions unclear.

Researcher considerations

1. Please detail the involvement that researchers who are based in the region(s) of study had during a) study design; b) clinical study processes, such as processing blood samples, prescribing medication, or patient recruitment; c) data interpretation; and d) manuscript preparation, commenting on all aspects. If they were not involved in any of these aspects, please explain why.

   This question is intended for international partnerships; if all your authors are based in the area of study, this question is not applicable.

   This should include a thorough description of their leadership role(s) in the study. Are local researchers named in the author list or the acknowledgements, or are they not mentioned at all (and, if not, why)? Please also describe the involvement of early career researchers based in the location of the study. Some of this information might be repeated from the Contributors section in the manuscript. Note: we adhere to ICMJE authorship criteria when deciding who should be named on a paper.

**a) Study design:**<br><br>This study was part of as multicounty Qualitative Longitudinal Study (QLS) in three African countries (Ghana, Kenya, and Malawi), designed as part of a collaboration between the Kenya Medical Research Institute (KEMRI, Kenya), the Institute of Health Research at the University of Health and Allied Sciences in Ghana (UHAS, Ghana), the College of Medicine at the University of Malawi (COM, Malawi), the Liverpool School of Tropical Medicine (LSTM) and the London School of Hygiene & Tropical Medicine (LSHTM) in the UK, the US Centers for Disease Control and Prevention (USA), and PATH (USA), the study sponsor.<br><br>This manuscript is one of three to be published from the study in Kenya. Kenyan researchers contributed significantly to the overall conception and design of the study which entailed substantial national/subnational tailoring to 'local' contexts.
**b) Clinical study processes:**

Not applicable.
**c) Data interpretation:**<br><br>Kenyan researchers contributed equally (to non-African researchers) to data interpretation for the study in Kenya. Data interpretation was a collaborative process and was conducted throughout the study as part of the QLS design. Regular discussion sessions between UK and Kenyan research team members were held during data collection and processing, coding and analysis as part of an iterative study design. At the end of the study, Kenyan and non-Kenyan researchers met to discuss and interpret study findings at a writing workshop with all members of the field research team. Three Kenyan co-authors gave substantial input into the analysis of the data for this manuscript.
**d) Manuscript preparation:**<br><br>Three members of the Kenyan research team, including the Kenya site PI and data manager, are co-authors and contributed to manuscript preparation. Other members of the team who contributed to data collection are named and acknowledged.<br><br>The research team met early on and at regular intervals throughout the duration of the wider study to discuss and develop manuscript ideas and authorship. As part of the wider Kenya study, at least one manuscript will be led by a Kenyan researcher.

2. Were the data used in your study collected by authors named on the paper, or have they been extracted from a source such as a national survey? ie, is this a secondary analysis of data that were not collected by the authors of this paper. If the authors of this paper were not involved in data collection, how were data interpreted with sufficient contextual knowledge?

   The Lancet Global Health *believe contextual understanding is crucial for informed data analysis and interpretation.*

The data used in our study was collected by authors named on the paper. Throughout the study, the Kenya team supported ongoing thematic analysis supported by the site PI and the UK PI and coder analyst to identify themes of relevance to feedback to the sub-national and national levels of the health system. During coding, themes identified by the coder analyst were discussed and verified by all members of the Kenya team. Observational data collected at the county, health facility levels and community levels collated by the Kenya team were used to enhance contextual understanding for analysis and interpretation.

3. How was funding used to remunerate and enhance the skills of researchers and institutions based in the area(s) of study? And how was funding used to improve research infrastructure in the area of study?

   Potentially effective investments into long-term skills and opportunities within institutions could include training or mentorship in analytical techniques and manuscript writing, opportunities to lead

all or specific aspects of the study, financial remuneration rather than requiring volunteers, and other professional development and educational opportunities.

Improvements to research infrastructure could be funding of extended trial designs (such as platform trials) and use of master protocols to enable these designs, establishment of long-term contracts for research staff, building research facilities, and local control of funding allocation.

**Skills:**<br>The project supported a Kenya post-doctoral position at KEMRI held by GO who was mentored by the study PI (JH) to become the study coordinator, responsible for all aspects of the study including staff recruitment, training and deployment, data management, coding and analysis and provided opportunities to present results at nationally and internationally convened meetings. GO received additional professional development support from his PhD supervisor at the KEMRI-Wellcome Trust Research Programme where he was affiliated. The post-doc and study PI were jointly responsible for building the capacity of the field research team on qualitative research methodologies, data processing, management, coding, analysis. Both GO and JH provided mentorship to the data manager to develop her data and project management skills, analytical skills and manuscript writing skills, and ensured her involvement in national MVIP meetings where she presented results.
**Research infrastructure:**<br><br>This project contributed to improvements in local infrastructure including computers, software, employment of local staff, transportation and recurrent costs for mobilising the research team and vehicle maintenance for field work.

4. How did you safeguard the researchers who implemented the study?

Please describe how you guaranteed safe working conditions for study staff, including provision of appropriate personal protective equipment, protection from violence, and prevention of overworking.

The safeguarding procedures used in this study were in line with the principals and guidelines on Safeguarding set out at KEMRI to protect research staff and participants. KEMRI has appointed Safeguarding lead who is responsible for training all research staff of safeguarding procedures, including producers for reporting safeguarding concerns. In addition, KEMRI operates strict working conditions, which prohibits travel after dark. All project related meetings took into consideration the time difference between the UK and Kenya and were kept within office hours. The study was suspended briefly during COVID, and a protocol amendment made to incorporate personal protection measures into data collection activities, as mandated by the Kenyan government.

Benefits to the communities and regions of study

5. How does the study address the research and policy priorities of its location?

How were the local priorities determined and then used to inform the research question? Who decided which priorities to take forward? Which elements of the study address those priorities?

Malaria remains a significant burden in the endemic regions in Kenya with cases of severe malaria occurring most frequently in children under 5 years. Despite implementation of effective preventive strategies, the decline in malaria deaths has stagnated and new strategies are needed to enhance efforts to further control malaria. After decades of research to develop a malaria vaccine, RTS,S/AS01 malaria vaccine emerged as a promising candidate offering a 40% reduction in malaria episodes and a significant reduction in life-threatening severe malaria caused by *P. falciparum*. Kenya was selected as one of three sub-Saharan African countries which applied to carry out the WHO-led pilot implementation of the new malaria vaccine and gather further evidence on the feasibility and uptake of the 4-dose schedule. Implementation of the malaria vaccine was overseen by the Malaria Vaccine Implementation Program (MVIP), comprising stakeholders at the global and country levels. In Kenya, the malaria vaccine introduction was led by the Ministry of Health's Immunisation and National Malaria Control programmes, and the nested evaluation studies received widespread support.

6. How will research products be shared in the community of study?

For instance, will you be providing written or oral layperson summaries for non-academic information sharing? Will study data be made available to institutions in the region(s) of study? The Lancet Global Health *encourages authors to translate the summary (abstract) into relevant languages after paper editing; do you intend to translate your summary?*

Prior to finalising all analyses the thematic findings were shared through presentations made to the health facilities, subcounties and counties (convened by county) and national level stakeholders for validation and input. Dissemination to national MOH and stakeholders was done through the monthly MVIP meetings. The study findings of the QLS (of which this manuscript is a component) have provided key insights and lessons learned that have been shared to help optimise the implementation and national roll out of the malaria vaccine in Kenya.

7. How were individuals, communities, and environments protected from harm?

a) *How did you ensure that sensitive patient data was handled safely and respectfully? Was there any potential for stigma or discrimination against participants arising from any of the procedures or outcomes of the study?*

None.

b) *Might any of the tests be experienced as invasive or culturally insensitive?*

Not applicable.

c) *How did you determine that work was sensitive to traditions, restrictions, and considerations of all cultural and religious groups in the study population?*

Not applicable.

d) *Were biowaste and radioactive waste disposed of in accordance with local laws?*

Not applicable.

e) *Were any structures built that would have impacted members of the community or the environment (such as handwashing facilities in a public space)? If so, how did you ensure that you had appropriate community buy-in?*

Not applicable.

f) *How might the study have impacted existing health-care resources (such as staff workloads, use of equipment that is typically employed elsewhere, or reallocation of public funds)?*

Interviews were conducted at a time and location chosen by the study participants.

8. Finally, please provide the title (eg, Dr/Prof, Mr/Mrs/Ms/Mx), name, and email address of an author who can be contacted about this statement. This can be the corresponding author.

Name: Dr George Okello
Email: gokello2002@gmail.com
